# Excellence in Rehabilitation Award

**DOI:** 10.1093/geroni/igaa057.3207

**Published:** 2020-12-16

## Abstract

The Excellence in Rehabilitation of Aging Persons Award lecture will feature an address by the 2020 Excellence in Rehabilitation Award recipient Kenneth J. Ottenbacher, PhD, OTR. The Excellence in Rehabilitation of Aging Persons Award is designed to acknowledge outstanding contributions in the field of the rehabilitation of aging individuals.

